# Topical ruxolitinib for treatment-induced vitiligo in a patient with metastatic melanoma

**DOI:** 10.1016/j.jdcr.2024.11.002

**Published:** 2024-11-26

**Authors:** Alpaslan Tasdogan, Dirk Schadendorf, Selma Ugurel

**Affiliations:** Department of Dermatology, University Hospital Essen, University Duisburg-Essen, German Cancer Consortium (DKTK), Partner site Essen/Düsseldorf, Essen, Germany

**Keywords:** adverse events, JAK inhibitor, melanoma, target therapy, vitiligo

## Introduction

Vitiligo is a well-known side effect in patients undergoing treatment for melanoma with either anti-PD-1 immunotherapy or B-Raf (BRAF)/mitogen-activated protein kinase (MEK)-directed targeted therapy.^1^ Its occurrence is related to a favorable tumor response and prolonged survival.^2^^,^^3^ Spontaneous repigmentation is occasionally observed in association with tumor relapse.^4^ Otherwise, the depigmentation persists and may cause distress to the patients concerned.

Here, we report a case of extensive vitiligo induced by adjuvant BRAF/MEK-inhibiting targeted therapy, which demonstrated strong repigmentation with topical ruxolitinib. To the best of our knowledge, this is the first report on the efficacy of topical ruxolitinib for treatment-induced vitiligo in melanoma.

## Case report

**Fig 1:**
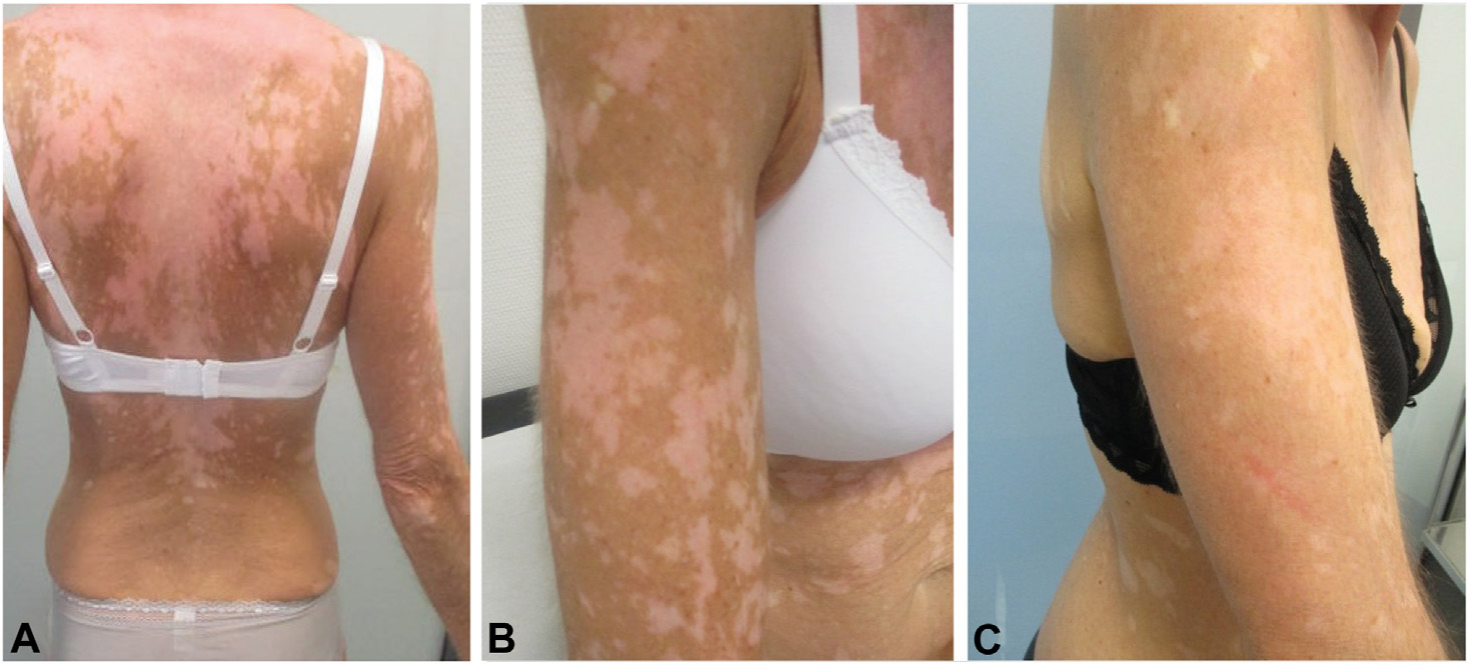
Vitiligo induced by adjuvant B-Raf/mitogen-activated protein kinase-directed therapy with dabrafenib plus trametinib (**A** and **B**), presenting strong repigmentation after 6 months of topical ruxolitinib (**C**).

A 56-year-old female who had been diagnosed with melanoma of the left cheek (tumor invasion depth of 0.65 mm; no ulceration; pT1a by American Joint Committee on Cancer [AJCC] v8), 4 years ago, presented in November 2021 at our dermatology department with a retroauricular painless swelling. The computed tomograpghy (CT) and magnetic resonance imaging (MRI) staging examinations did not reveal any additional tumor lesions. Surgery and histopathology revealed a single lymph node macrometastasis, BRAF-mutated (V600 R), and PD-L1 expression positive (cut -off 5%). In January 2022, adjuvant therapy with the BRAF/MEK-inhibitors dabrafenib plus trametinib was initiated. The patient reported a number of adverse side effects, including fatigue, febrile episodes, headache and joint pain, as well as vitiligo. The severity of all side effects, with the exception of the vitiligo, demonstrated a decline within the initial 6-month period of ongoing treatment. Vitiligo began in the fourth month after initiation of adjuvant therapy and initially affected the chest and upper back. There was a progressive increase in the extent of the condition throughout the course of ongoing treatment. After the scheduled end of treatment with dabrafenib plus trametinib in January 2023, the extent of vitiligo on the patient’s body surface increased further, with the trunk and upper extremities as well as the patient’s face becoming largely affected (Fig 1, *A* and *B*). Given the significant impact of progressive vitiligo on the patient’s quality of life, we initiated topical monotherapy of the affected skin areas with the Janus kinase (JAK) inhibitor ruxolitinib. After 3 months of twice-daily ruxolitinib (15 mg/g) administration, erythema was observed in the treated areas, followed by significant repigmentation after 6 months of ruxolitinib (Fig 1, *C*).

## Discussion

Vitiligo affects approximately 0.5% to 2% of the global population, and can significantly impact the patient’s quality of life due to its visible nature, although it is not life-threatening. The disease is often associated with a considerable psychosocial burden, including stigma and emotional distress. Etiopathogenically, the patient’s immune system mistakenly targets melanocytes, leading to characteristic hypopigmented patches on the skin. The melanocyte destruction can be mediated by different immune mechanisms, including autoreactive cytotoxic T cells and autoantibodies targeting melanocyte-specific antigens, both relying on cytokine signaling pathways.

Recent preclinical and translational studies have demonstrated that interferon-γ signaling through the JAK pathway is a significant pathogenic driver of the disease.^5^ Thus, ruxolitinib has been gaining attention for its use in treating non-segmental vitiligo. By inhibiting JAK1 and JAK2, ruxolitinib helps to reduce inflammation and potentially restore pigment to the affected areas. In 2 phase-3 trials, topical application of ruxolitinib twice daily for 24 weeks resulted in repigmentation of vitiligo lesions of at least 75% from baseline in approximately 30% of patients.^6^ In this study, topical ruxolitinib was well tolerated with the most common adverse events being acne and pruritus at the application sites.^6^ In our patient, we did not observe any of these side effects with topical ruxolitinib treatment.

Vitiligo is widely recognized as a common side effect of immunotherapy. With a correlation between the onset of vitiligo and treatment outcome, it underscores the complex interplay between cancer treatment and immune response.^7^ However, evidence of correlation between vitiligo and the efficacy of BRAF/MEK-directed targeted therapy is rare. It may be attributed to the fact that certain targeted therapies enhance the immune system's ability to recognize and destroy melanoma cells. However, they may also inadvertently attack normal melanocytes, leading to vitiligo. While it can be a sign of effective therapy, it also necessitates the implementation of supportive care to address the cosmetic and psychological impact on patients. At our melanoma center, we often observe that upon treatment with BRAF/MEK inhibitors, vitiligo develops and progresses during treatment, and persists even after the end of treatment. In these patients, extensive vitiligo can significantly affect mental health, leading to depression, anxiety, and social isolation. Although oral ruxolitinib has the potential to exert systemic immunosuppressive effects, topical ruxolitinib is confined to the skin and its systemic absorption is significantly lower.^8^ Therefore, the probability of interference with immunotherapy is reduced when ruxolitinib is administered topically.^8^ At present, there is lack of clinical data that has been specifically designed to assess the interaction between topical ruxolitinib and cancer therapies, including checkpoint inhibitors and BRAF/MEK inhibitors.

Our case demonstrates that JAK inhibition by ruxolitinib is effective in improving treatment-induced vitiligo. Further research is needed to better understand this phenomenon and improve management strategies for those affected.

## Conflicts of interest

None disclosed.
